# Silent Circulation of the Saint Louis Encephalitis Virus among Humans and Equids, Southeast Brazil

**DOI:** 10.3390/v11111029

**Published:** 2019-11-05

**Authors:** Galileu Barbosa Costa, Paula Eillany Silva Marinho, Ana Paula Pessoa Vilela, Ana Teresa Saraiva-Silva, Ana Paula Correia Crispim, Iara Apolinário Borges, Ana Gabriella Stoffella Dutra, Zélia Inês Portela Lobato, Jenner Karlison Pimenta dos Reis, Danilo Bretas de Oliveira, Betania Paiva Drumond, Erna Geessien Kroon, Giliane de Souza Trindade

**Affiliations:** 1 Laboratório de Vírus, Departamento de Microbiologia, Instituto de Ciências Biológicas, Universidade Federal de Minas Gerais. Avenida Presidente Antônio Carlos, 6627, Pampulha, Belo Horizonte, Minas Gerais 31270901, Brasil; ei_lanny@hotmail.com (P.E.S.M.); pessoavilela58@gmail.com (A.P.P.V.); anateresasaraivasilva@gmail.com (A.T.S.-S.); anapbio2@gmail.com (A.P.C.C.); borges2805@gmail.com (I.A.B.); anagstoffella@gmail.com (A.G.S.D.); betaniadrumond@gmail.com (B.P.D.); ernagkroon@gmail.com (E.G.K.); 2 Laboratório de Pesquisa em Virologia Animal, Departamento de Medicina Veterinária Preventiva, Escola de Veterinária, Universidade Federal de Minas Gerais, Belo Horizonte, Minas Gerais 31270901, Brazil; ziplobato@gmail.com; 3 Laboratório de Retroviroses, Departamento de Medicina Veterinária Preventiva, Escola de Veterinária, Universidade Federal de Minas Gerais, Belo Horizonte, Minas Gerais 31270901, Brazil; jennerkpr@gmail.com; 4 Faculdade de Medicina, Universidade Federal dos Vales do Jequitinhonha e Mucuri, Diamantina 39100000, Brazil

**Keywords:** Saint Louis encephalitis virus, seroprevalence, epidemiology, asymptomatic cases, public health burden

## Abstract

Saint Louis encephalitis virus (SLEV) is a mosquito-borne flavivirus that occurs throughout the Americas, and is considered a public health threat. In Brazil, SLEV has been detected from human cases associated with dengue-like disease, but no neurological symptoms were reported. Furthermore, the epidemiology of SLEV in human populations is still poorly explored in the country. We reported serological and molecular detection of SLEV in a healthy population of equids and humans from rural areas in Southeast Brazil. A plaque reduction neutralization test was applied, and neutralizing antibodies were detected in 11 individuals (4.6%) and 60 horses (21.5%). A qPCR targeting the 5′UTR region and reverse transcription-PCR (RT-PCR) targeting the non-structural protein (NS5) gene were performed and three individuals tested positive in both assays. Subsequent phylogenetic analysis confirmed SLEV circulation and its findings suggest the occurrence of an asymptomatic or subclinical presence in human and animal cases, correlating with the risks for outbreaks and consequently burden of SLEV infections to public health. Preventive strategies should include improved surveillance in regions with a high probability of SLEV occurrence, improvement in diagnostic methods, and evaluation of exposure/risk factors that can favor SLEV emergence.

## 1. Introduction

Saint Louis encephalitis virus (SLEV) is a mosquito-borne virus that causes human and animal encephalitis in the Western hemisphere [1]. SLEV is a member of the *Flavivirus* genus (*Flaviviridae* family), which include West Nile virus, Japanese encephalitis virus, Dengue virus, and Yellow fever virus [2].

SLEV is maintained in a zoonotic cycle, where birds are the natural amplifying host, with other vertebrates (involving equids and humans) considered accidental hosts. Human infections with SLEV are mostly asymptomatic in which infected individuals present mild malaise or flu-like symptoms. Severe cases are clinically characterized by high fever, neurological dysfunction, altered consciousness, and headaches, which are accompanied by encephalitis or meningoencephalitis [3,4].

In Brazil, SLEV circulation has been reported in Southeast and Midwest regions in the past 13 years, mostly presenting mild symptoms, related to suspected dengue cases, and most infections being misdiagnosed [5,6,7,8,9,10]. Serological studies showed SLEV circulation among horses from different regions of Brazil [11,12] and furthermore, SLEV was isolated from a horse with neurological disease in the state of Minas Gerais, Southeast Brazil. This data altogether highlights the potential spread and the risk of possible outbreaks caused by SLEV in Brazil [13]. Hence, the primary objective of this study was to investigate whether equids and humans from rural areas in Southeast Brazil have had evidence of exposure to SLEV.

## 2. Materials and Methods

We retrospectively analyzed 279 equid serum samples collected between 2003–2004, and 2011–2012 in the Southeast region of Brazil. The equids samples were obtained from a large study related to the investigation of the incidence of Orthopoxvirus in Minas Gerais [14]. Sera samples were collected from different regions (mesoregions) around Minas Gerais such as (1) Triângulo Mineiro and Alto Parnaíba (70 samples); (2) Central Mineira and Centro-Oeste de Minas (71 samples); (3) Campo das Vertentes and Zona da Mata (49 samples); (4) Vale do Rio Doce (14 samples); (5) Vale do Jequitinhonha and Mucuri (75 samples). Equids from rural areas of Serro city were sampled in October 2012, during the rainy season. Equids from the other mesoregions were sampled during both dry and rainy seasons.

**Figure 1:**
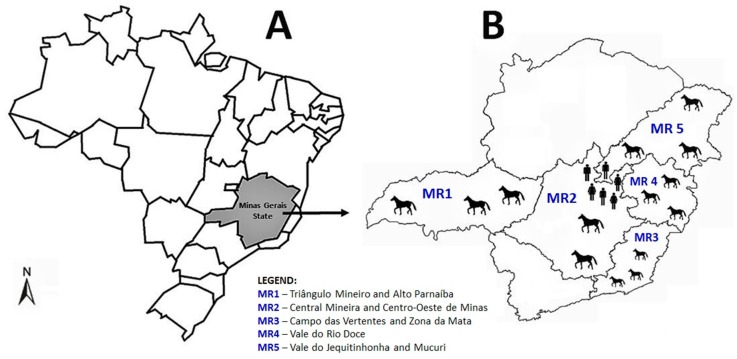
An overview of the studied area. (**A**) Map of Brazil highlighting the state of Minas Gerais in grey, Southeast region of the country. (**B**) A detailed map of Minas Gerais showing the regions where equids and humans were sampled in this study. The regions are divided in seven subregions (meroregions (MR)) according to the animal defense bureau of Minas Gerais.

An epidemiological questionnaire was carried out during the sampling through an on-farm interview with the owners, in order to obtain data related to the animals (Table 1). The explanatory variables collected were age, gender, breed, and region. We further decided to evaluate 240 human serum samples from one of the areas (Serro region, Figure 1). The human samples were also obtained from a study carried out to investigate the prevalence of Orthopoxvirus in that region [15]. Individuals from rural areas of Serro city were enrolled during September 2012 to March 2013, which is the rainy season. A structured questionnaire was applied to collect the following demographic information: age, gender, self-reported skin color, income, and educational level. Information on contact with equids and wild environment were also included (Table 2).

It is important to emphasize that Minas Gerais is in three different biomes: The Cerrado (South American Savannah), covering 50% of the State; Atlantic Forest, and Caatinga (desert vegetation) located in the North part of the State [16,17]. Serro city is characterized by a transition area between the Cerrado and Atlantic Forest, with a subtropical climate and two well-defined seasons, a dry season (between May and September) and a rainy season (between October and April) [16,17].

Although there is no data describing the occurrence or abundance of *Culex* species in the study area so far, studies conducted in the Amazon area highlighted *Culex* species (*Culex coronator* and *Culex declarator*) frequently found infected with SLEV [18,19], which suggests these vectors could play an important role in wild transmission cycle of SLEV. It is also important to say that SLEV was already isolated from *Culex quinquefasciatus* in Argentina, suggesting this mosquito may play an important role as a vector in the urban transmission cycle of SLEV [20,21].

To determine the presence of neutralizing antibodies in equid sera samples, we used the plaque reduction neutralization test (PRNT) [22]. Serum samples were initially heated in a water bath at 56 °C for 30 min to denature complement system proteins and subsequently diluted in Eagle’s minimum essential medium (MEM) free of fetal bovine sera (FBS) to a screening ratio of 1:20. Samples were added to the same volume (1:1) of a solution containing approximately 100 plaque forming units (PFU) of SLEV (strain BeH 355964) diluted in FBS free MEM. The final solution (virus/serum) was homogenized and incubated for 1 h at 37 °C. Six-well plates containing VERO cells monolayers with 100% confluence were inoculated with virus/serum solution and incubated at 37 °C for 1 h in an atmosphere supplemented with 5% of CO_2_. Later, MEM supplemented with 2% FBS and 1.5% carboxymethyl cellulose was added to each well and plates were incubated for five days at 37 °C in atmosphere supplemented with 5% of CO_2_. VERO monolayers were then fixed with formalin at 10% and stained with crystal violet solution at 1%. All samples were tested in duplicate. Positive samples were defined as the highest dilution that inhibited ≥90% of virus plaques compared with negative controls. All positive samples were titrated according to the PRNT protocol described above, and the last dilution in which ≥90% PFU reduction was observed was used as a reference to calculate the value of neutralizing units per milliliter. The value was obtained by dividing 1 mL by the volume of virus/serum solution inoculated and multiplying it by the last positive dilution value.

To investigate the presence of SLEV RNA in sera samples, we performed qPCR targeting the 5′UTR region and a multiplex nested reverse transcription-PCR (RT-PCR) targeting the non-structural protein (NS5) gene [23]. The qPCR was performed with a commercial mix (SYBR^®^ Green PCR Master Mix, Life Technologies, Carlsbad, CA, USA). Briefly, a denaturation cycle at 95 °C for 10 min, followed by 45 cycles of 95 °C for 15 s and 60 °C for 1 min. Finally, a dissociation curve with heating from 65 °C to 95 °C. A specific amplification with a melting temperature of 79.5 °C was also included. The primers used for amplification were forward 5′–CAGGGAATTACCCAATGTCTAAAAA–3′, and reverse 5′–AGCATATTGACAACCCGGTTTC–3′.

We directly sequenced the RT-PCR amplified fragments in both orientations and in duplicate by using the ABI3130 platform (Applied Biosystems, Foster city, CA, USA). We used ClustalW (http://www.genome.jp/tools/clustalw/) and MEGA7 software (http://megasoftware.net/) to align nucleotide sequences and construct a phylogenetic tree by using the maximum-likelihood method.

The Pearson Chi-squared and Fisher’s exact tests with a significance level of 5% using Epi Info^TM^ software, version 7.2.1.0. (www.cdc.gov/epiinfo) were applied to verify the statistical significance between the serological results and studied variables that could be related to SLEV circulation. Relative odds ratios and 95% confidence intervals were calculated when applied.

This study was approved by the Research Ethics Committee on Animal Experimentation under the protocol 131/2010, and by the Human Research Ethics Committee of Universidade Federal de Minas Gerais under the protocol FR–413704.

## 3. Results

We detected anti-SLEV neutralizing antibodies in 60 equids (prevalence rate of 21.5%; 95% CI: 17.1–26.7%) (Table 1), with antibodies titers ranging from 100 to 300 neutralizing units (NU)/mL. Interestingly, most seropositive equids (*n* = 33, 55%) were sampled during 2003–2004. A total of 17 seropositive equids were sampled in 2011 (*n* = 17, 28.3%) and 10 (16.7%) in 2012. Equids aged between 4–8 years old were almost three times more likely to have neutralizing antibodies than younger equids. Moreover, equids from Triângulo Mineiro and Alto Parnaíba (MR1), Central Mineira and Centro-Oeste de Minas (MR2), and Vale do Jequitinhonha and Mucuri (MR5) were more likely to have neutralizing antibodies compared to equids from Campo das Vertentes and Zona da Mata (MR3) and Vale do Rio Doce (MR4) (Table 1).

We also detected 11 seropositive individuals (prevalence rate of 4.6%; 95% CI: 2.5–8.1%) (Table 2), with antibodies titers also ranging from 100 to 300 NU/mL. No variables related to the human population were significantly associated with the presence of neutralizing antibodies (Table 2). However, it is important to highlight that 141 individuals (58.8%) reported contact with equids.

**Figure 2:**
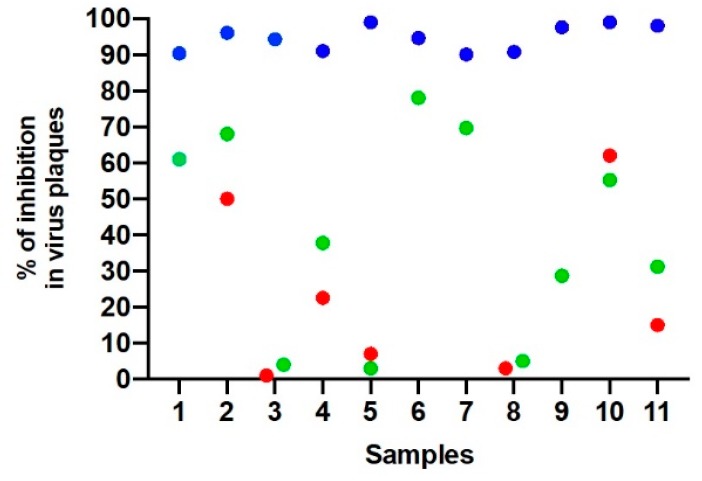
Relationship among neutralizing antibodies against Saint Louis encephalitis virus (SLEV), YFV, and Dengue virus serotype 4 (DENV-4) detected in 11 human serum samples from the state of Minas Gerais, Brazil, 2011–2012. Blue dots: Serum samples that exhibited ≥90% reduction in virus plaques for SLEV; Green dots: serum samples that exhibited 3–62% reduction in virus plaques for YFV. Red dots: serum samples that exhibited 3.7–70% reduction in virus plaques for DENV4. Samples 1, 6, 7, and 9 were not tested for neutralizing antibodies against YFV.

As the studied area has a high risk for Yellow Fever occurrence and vaccination is strongly recommended [24], a complementary ≥90% PRNT assay was performed against Yellow Fever virus (YFV) strain 17D to rule out any cross-reactive antibody response with the yellow fever vaccine. A total of seven human serum samples that exhibited ≥90% of reduction in virus plaques for SLEV had neutralizing antibodies against YFV with reduction ranging from 3% to 62% (Figure 2). However, it was not possible to test neutralizing antibodies against YFV in four of eleven samples. We also performed another complementary ≥90% PRNT assay against Dengue virus serotype 4 (DENV-4), and the 11 human serum samples had neutralizing antibodies with reduction ranging from 13.7% to 70% (Figure 2).

**Figure 3:**
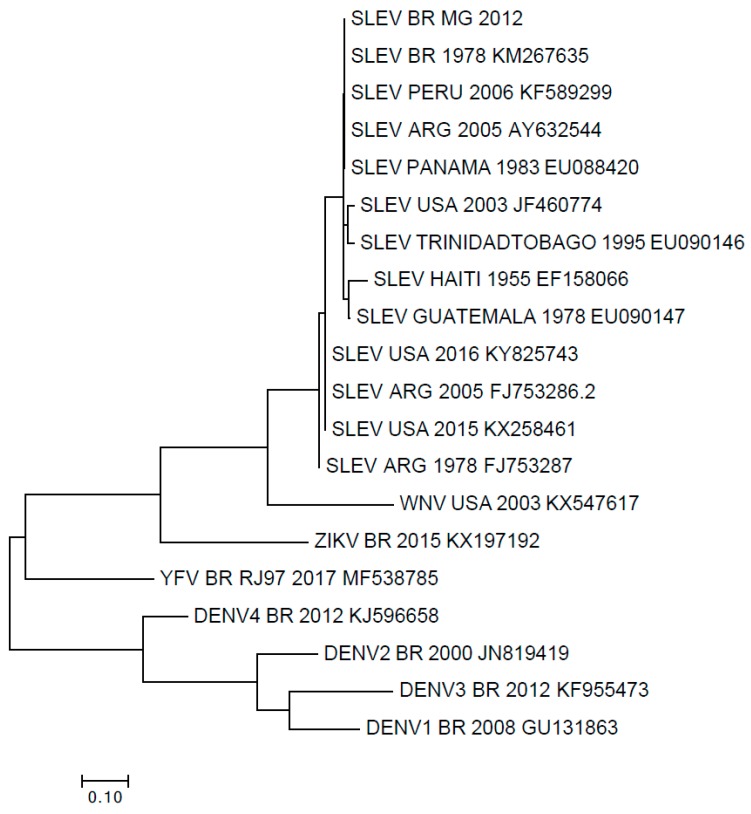
Phylogenetic tree based on nucleotide sequences of the 5′UTR region of Flaviviruses. The evolutionary history was inferred by using the maximum likelihood method based on the Tamura-Nei model. The tree with the highest log likelihood (–712.38) is shown. Initial tree(s) for the heuristic search were obtained automatically by applying neighbor-join and BioNJ algorithms to a matrix of pairwise distances estimated using the maximum composite likelihood (MCL) approach, and then selecting the topology with superior log likelihood value. The tree is drawn to scale, with branch lengths measured in the number of substitutions per site. The analysis involved 20 nucleotide sequences. Codon positions included were 1st + 2nd + 3rd + Noncoding. All positions containing gaps and missing data were eliminated. There was a total of 79 positions in the final dataset.

**Figure 4:**
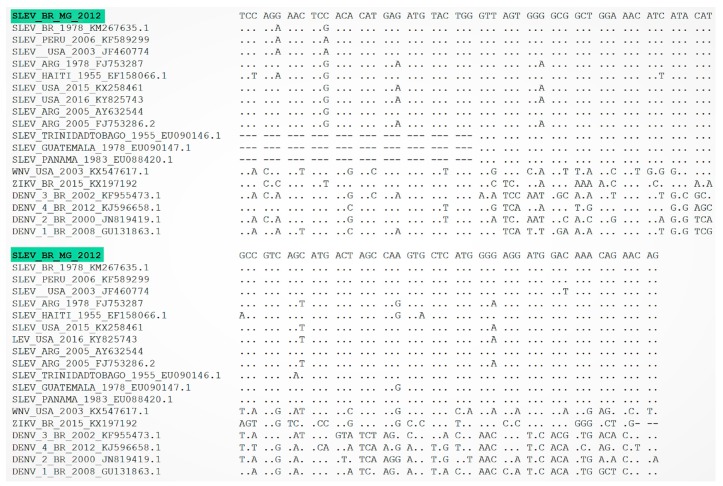
Nucleotide sequence of the SLEV 5′UTR region detected in humans (green) compared with homologous sequences of several other SLEV strains detected in North and Latin America. The sequenced sample showed two polymorphisms (G–A and C–G) in comparison with other SLEV isolates.

Overall, three human serum samples tested positive for the 5′UTR region in qPCR and NS5 gene in RT-PCR, yielding a nucleic acid detection rate of 1.25%. Sequences were grouped with SLEV isolates from Brazil, USA, and South America (Figure 3 and Figure 4). Upon phylogenetic analysis, the detected SLEV strain clustered within a group that included SLEV strains detected in South and Central America (Figure 3 and Figure 4). To rule out infection with Dengue virus, a complementary RT-PCR was also performed [23], and the tested human serum samples had negative results.

## 4. Discussion

In the present study, we assessed SLEV exposure among equids and humans from Southeast Brazil. During recent decades, a considerable number of arboviruses have been detected in the country [10,12]. Taking into account the emergence and reemergence of medically important arboviruses such as *Dengue, Chikungunya, Yellow fever,* and *Zika viruses, the active surveillance and epidemiological characterization of other circulating arboviruses into the country is necessary, due to the nature of infection and associated outcomes*.

The seroprevalence of SLEV in equine populations has been already described in Brazil, which ranges from 12.3% to 22.6% [11,12]. Our findings revealed a higher seroprevalence during 2003–2004, although no SLEV outbreaks or occurrence have been reported around that time. However, the seroprevalence dropped on equids sampled during 2011–2012, corroborating results from Silva and colleagues who found 20.0% of seropositive equids from the same region we studied [11].

Regarding the seroprevalence of SLEV in human populations, according to the literature it ranges from 3.1% to 7.1% [25,26], which is not far from our findings (4.6%), but lower than the seroprevalence in Argentina, ~14.0% [27,28]. Considering that SLEV infections are probably not rare in Brazil (especially in the Southeast region and neighbor states), and cases could remain undiagnosed or misinterpreted, the enhancement of a surveillance system and diagnostic tools are needed. Furthermore, the human population included in this study could represent asymptomatic cases, reinforcing the hypothesis of silent SLEV circulation in the Southeast area of Brazil.

Although flaviviruse infections produce short-lived viremia in serum (up to 7 days post onset of symptoms), and the detection in clinically unwell patients is difficult, we were able to detect three of 240 positive human serum samples in RT-PCR and qPCR assays. Further sequencing of 5′UTR amplicons confirmed the presence of a SLEV strain similar to other strains previously detected in Brazil. Recently, Rivalora and colleagues showed that Swiss albino mice infected with a SLEV strain isolated during an epidemic period in Argentina produced higher viremia levels when compared to infected mice with a non-epidemic strain [29]. This study also corroborates a study conducted by Monath et al. (1980) that also detected higher viremia levels in Swiss albino mice infected with an epidemic SLEV strain [30]. Furthermore, it is important to emphasize that the host immune system could also contribute to the rapid elimination of viral particles.

Our study has limitations. We were unable to establish a comparative titer for Brazilian flaviviruses other than YFV and DENV-4 due to the insufficient volume of serum samples. However, the PRNT is the gold standard test for serological diagnosis and confirmation due to its high sensitivity and specificity for distinguishing any of the arthropod-borne flaviviruses [31,32]. A PRNT test with ≥90% inhibition of virus plaques presents better distinction due to the low cross-reactivity level when compared with a PRNT with 50% inhibition [31,32]. As the human samples were collected to understand the seroprevalence of Orthopoxvirus in the Serro region, we did not undertake specific questions or characteristics of the population that are associated with Flavivirus occurrence, which could have introduced bias to this study. In addition, the equid samples were not fully tested in the molecular assays due to the serum samples being stored at a minimum of −20°C, therefore compromising the RNA preservation and consequently its detection. Due to this complication, we were not able to obtain a greater sequence and the sequenced fragment was not enough to determine phylogenetic inferences. However, the data presented here contributes to the knowledge of SLEV epidemiology in previously unstudied areas, showing a broader distribution in rural areas of Brazil.

## 5. Conclusions

In conclusion, the data presented here demonstrates a possible silent SLEV circulation in Brazil, with potential asymptomatic cases remaining undiagnosed. Additionally, the epidemiology of SLEV among human and animal populations is still poorly explored, highlighting the importance of an effective surveillance. As suggested by Figueiredo, protocols for the molecular diagnostics of flavivirus infections including SLEV should be addressed [10]. Further research is necessary to have a better understanding of the epidemiology of SLEV in Brazil, monitoring its transmission, and helping professionals in the field with early interventions to reduce the burden of related cases.

## Figures and Tables

**Table 1:** Characteristics of equid population related to neutralizing antibodies against Saint Louis encephalitis virus, Minas Gerais, Brazil, 2011–2012.

Variables	Equid Population *n* = 279 (%)	Seropositive *n* = 60 (%)	Seronegative *n* = 219 (%)	Odds Ratio 95% CI	*p* Value
**Sex**					
Female	104 (37.3)	28 (46.7)	76 (34.7)		Reference
Male	175 (62.7)	32 (53.3)	143 (65.3)	1.6 (0.9–3.0)	0.1
**Age (years)**					
≤3	56 (20.1)	6 (10.0)	50 (22.8)		Reference
4–8	137 (49.1)	35 (58.3)	102 (46.6)	2.9 (1.1–7.2)	0.03
>8	86 (30.8)	19 (31.7)	67 (30.6)	0.4 (0.1–1.1)	0.1
**Breed**					
*Equus cabalus* *	258 (92.5)	54 (90.0)	204 (93.2)		Reference
Hibrids **	21 (7.5)	6 (10.0)	15 (6.8)	0.7 (0.2–1.8)	0.6
**Region ^†^**				
MR1	70 (25.1)	13 (21.7)	57 (26.0)	5.8 (1.7–19.8)	0.01
MR2	71 (25.4)	12 (20.0)	59 (26.9)	6.5 (1.9–22.4)	0.006
MR3	49 (17.6)	18 (30.0)	31 (14.1)	2.3 (0.7–7.7)	0.03
MR4	14 (5.0)	8 (13.3)	6 (2.7)	9.8 (2.7–34.7)	0.001
MR5	75 (26.9)	9 (15.0)	66 (30.1)		Reference

* *Equus cabalus* = stallions and mares. ** Hibrids = mules. ^†^ MR1 = Triângulo Mineiro and Alto Parnaíba; MR2 = Central Mineira and Centro-Oeste de Minas; MR3 = Campo das Vertentes and Zona da Mata; MR4 = Vale do Rio Doce; MR5 = Vale do Jequitinhonha and Mucuri.

**Table 2:** Characteristics of the human population related to neutralizing antibodies against Saint Louis encephalitis virus, Minas Gerais, Brazil, 2011–2012.

Variables	Human Population *n* = 240 (%) *	Seropositive *n* = 11 (%)	Seronegative *n* = 229 (%)	Odds Ratio 95% CI	*P* Value
**Gender**					
Female	113 (47.1)	4 (36.4)	106 (46.3)	0.67	Reference
Male	127 (52.9)	7 (63.6)	123 (53.7)	(0.19–2.32)	0.7
**Age (years)**					
≤18	40 (16.7)	2 (18.2)	38 (16.6)	***	Reference
19–30	40 (16.7)	0	40 (17.5)		0.7
31–50	97 (40.4)	4 (36.4)	93 (40.6)		0.99
>50	63 (26.2)	5 (45.4)	58 (25.3)		0.9
**Years of schooling completed**					
None	24 (10.0)	0	24 (10.5)	***	Reference
≤6	156 (65.0)	9 (81.8)	147 (64.2)		0.99
7–12	57 (23.8)	2 (18.2)	55 (24.0)		0.99
>12	3 (1.2)	0	3 (1.3)		0.99
Income **					
≤1 min wage	178 (74.2)	11 (100.0)	167 (72.9)	2.5	Reference
>1 min wage	19 (7.9)	0	19 (8.3)	(0.14–44.3)	0.99
**Contact with equids**					
Yes	141 (58.8)	7 (63.6)	134 (58.5)	1.2	0.99
No	96 (41.2)	4 (36.4)	92 (41.5)	(0.3–4.2)	Reference
**Contact with wild environment**					
Yes	117 (48.8)	8 (72.7)	109 (47.6)	2.9	0.2
No	123 (51.2)	3 (27.3)	120 (52.4)	(0.75–11.3)	Reference

* Total may not add up to 100% due to missing information. ** Income value in Brazilian currency in 2012 = R$ 622.00 (R$ 1.00 = US$ 2.08 approximately). ***Odds Ratio not calculated.
